# Analysis of factors influencing the clinical outcome after surgery and ^131^I therapy in patients with moderate-risk thyroid papillary carcinoma

**DOI:** 10.3389/fendo.2022.1015798

**Published:** 2022-10-14

**Authors:** Yixuan Li, Maohua Rao, Chenxi Zheng, Jiahui Huang, Danzhou Fang, Yalan Xiong, Gengbiao Yuan

**Affiliations:** Department of Nuclear Medicine, The Second Affiliated Hospital of Chongqing Medical University, Chong Qing, China

**Keywords:** papillary thyroid carcinoma, ^131I^ treatment, thyroglobulin, treatment response, excellent response

## Abstract

**Purpose:**

Generally, the prognosis for papillary thyroid cancer (PTC) is favorable. However, the moderate risk involved warrants further evaluation. Hence, we investigated the clinical outcomes in patients with moderate-risk PTC following surgery and the first ^131^I therapy, as well as the relevant factors that influence the therapeutic efficacy.

**Methods:**

Retrospective analyses of 175 patients with medium-risk PTC who visited the Second Affiliated Hospital of Chongqing Medical University from September 2017 to April 2019 were conducted. In according with the 2015 American Thyroid Association (ATA) guideline treatment response evaluation system, the patients were categorized into the following groups: excellent response (ER), indeterminate response (IDR), biochemical incomplete response (BIR), and structurally incomplete response (SIR), of which IDR, BIR, and SIR were collectively referred to as the NER group. To compare the general clinical features between the 2 groups of patients, 2 independent samples *t-*tests, *χ^2^* test, and Mann–Whitney *U-*test were performed, followed by multivariate logistic regression analyses. With reference to the receiver operating characteristic (ROC) curve, the predicted value of ps-Tg to ER was evaluated, and the best cut-off value was determined. The subgroups with BRAF^V600E^ test results were analyzed by *χ*2 test only.

**Results:**

The treatment responses of 123 patients were ER, while those of 52 patients were NER. The differences in the maximum tumor diameter (*U =* 2495.50), the amount of metastatic lymph nodes (*U =* 2313.50), the size of metastatic lymph node (*U* = 2113.50), the metastatic lymph node ratio (*U* = 2111.50), metastatic lymph node location (*χ^2^* = 9.20), and ps-Tg level (*U =* 1011.00) were statistically significant. Multivariate regression analysis revealed that ps-Tg (OR = 1.209, 95% *CI*: 1.120–1.305) was an independent variable affecting ER. The cut-off value of ps-Tg for predicting ER was 6.915 ug/L, while its sensitivity and specificity were 69.2% and 89.4%, respectively.

**Conclusions:**

Patients with smaller tumor size, fewer lymph nodes, lower metastatic lymph node ratio, metastatic lymph nodes in the central region, smaller lymph node size, and ps-Tg <6.915 ug/L demonstrated better therapeutic effects after the initial treatment.

## Introduction

The most common pathogenic type of thyroid carcinoma is papillary thyroid carcinoma (PTC), which has been associated with relatively low disease-specific mortality and excellent overall survival (1). Post-operative ^131^I therapy is an essential adjuvant therapy that can dramatically reduce the recurrence rate and occurrence of death in patients with differentiated thyroid cancer (DTC) (2). Presently, the majority of the domestic and international studies are focused on validating the clinical application of recurrence risk and the clinical significance of predicting disease recurrence or persistent disease, whereas the factors influencing the achievement of different clinical regression after the first ^131^I treatment for thyroid cancer after surgery are not well documented or in consensus (3).

Recent reports suggest that patients with DTC in the same risk stratification vary greatly in terms of their response to treatment, and it has been reported that patients with PTC who show an initial recurrence risk level of intermediate risk can be reclassified in the low-risk group of patients with low postoperative thyroglobulin (Tg) levels (4). Radioactive iodine (RAI) residual ablation is not routinely advised for American Thyroid Association (ATA) low-risk DTC patients (5) following thyroidectomy, and the factors influencing the accomplishment of various test results after ^131^I therapy in intermediate-risk patients have rarely been reported in the literature. With reference to the original 2009 ATA (6) system, microscopic tumor invasion to the perithyroidal soft tissues, RAI-avid metastatic foci in the neck detected on the first post-treatment whole-body RAI scan, aggressive histology, and PTC with lymphovascular invasion all categorized as intermediate hazards. The 2015 ATA (7) risk stratification added the risk factors for 5 clinical node positivity (cN1) or pathological N1 with >5 LNs (all involved LNs <3 cm in the largest dimension) and multifocal papillary microcarcinoma with *ETE* and *BRAF^V600E^* mutation (if known).

This survey was combined with the 2015 ATA recurrence risk stratification and treatment response assessment system to investigate the clinical regression of individuals with intermediate-risk PTC after surgery and ^131^I treatment, as well as the factors that influence it. The results provide a foundation for early assessment and personalized treatment of intermediate-risk PTC patients, which offers some guiding assistance.

## Materials and methods

### Ethics statement

The Second Affiliated Hospital of Chongqing Medical University’s Ethics Committee evaluated and authorized the experiments using human subjects while adhering to the principles of the Helsinki Declaration.

### Patients

A total of 175 intermediate-risk patients were included in this retrospective examination, which included 371 intermediate-risk PTC patients who had visited the Second Hospital of Chongqing Medical University from September 2017 to April 2019, with reference to the ATA guidelines and inclusion criteria. The subjects included 58 men and 117 women of age 42.1 ± 11.03 years, with a median follow-up time of 21 (18–49) months. The inclusion criteria included the following: ① pathologically confirmed diagnosis of PTC with bilateral total thyroidectomy and cervical lymph node dissection; ② first postoperative ^131^I treatment; ③ negative thyroglobulin antibodies (TgAb) (≤115 kU/L); ④ at least 6 months of follow-up with complete medical records; ⑤ meeting the 2015 ATA guidelines for moderate-risk stratification. The exclusion criteria included the following: ① distant metastasis; ② incomplete tumor resection; ③ macroscopically, the tumor infiltrated the thyroid’s soft tissues. We also referred to the 2015 ATA guidelines for moderate-risk stratification, as given below (meeting any of the following criteria was required) (8): ① microscopically, the tumor invaded the soft tissues surrounding the thyroid; ② first post-treatment whole-body RAI scan indicating RAI avid metastatic foci in the neck; ③ histology showing aggression (e.g., tall cell, hobnail variant, or columnar cell carcinoma); ④ PTC with vascular invasion; ⑤ clinical N1 or >5 pathologic N1 with all lymph nodes involved being <3 cm in the largest dimension; ⑥ multifocal papillary microcarcinoma with extrathyroidal extension and BRAF V600E mutated (if known); ⑦ intrathyroid, PTC, primary tumor 1–4 cm, *BRAF^V600E^* mutated (if known).TNM staging was classified the American Joint Commission for Cancer (8^th^ edition) AJCC (8) edition criteria.

### Methods

After thyroidectomy, the patients were treated with ^131^I (at the dose of 3.7 GBq) because we believed that most patients with DTC after thyroidectomy should receive a sufficient dose of ^131^I to destroy all normal residual thyroid tissue. This approach ensured that the serum thyroglobulin results monitored after treatment were relatively more reliable. under the thyroid-stimulating hormone (TSH) level >30 mU/L, and pre-ablation-stimulated Tg (ps-Tg) as well as TgAb were measured before ^131^I treatment, and cervical lymph nodes ultrasound, chest CT, head MRI, and other related examinations were also performed. A whole body scan (WBS) was performed a week after ^131^I treatment. ^131^I treatment was regular and followed by TSH inhibition therapy, while serological and imaging examinations were completed at the hospital 6 months later, which were also followed up regularly.

### Efficacy grouping

In accordance with the 2015 ATA guidelines (9), the subjects were classified based on their post-treatment response as follows: 1. Excellent response(ER): negative imaging *and either s*uppressed g <0.2 ug/L *or* stimulated Tg <1 ug/L (TgAb negative). 2. Indeterminate response(IDR): 0.2 μg/L ≤ suppressed Tg < 1 μg/L or 1 μg/L ≤ stimulated Tg <10 μg/L, TgAb stable or reduced. There was no evidence of radiographically confirmed structural or functional disease. After treatment, DX-WBS indicated weak imaging of the thyroid bed region. 3.Structural incomplete response(SIR): Tg or TgAb at any level and evidence of verifiable structural or functional disease. 4. Biochemical incomplete response(BIR): suppressed Tg ≥ 1 μg/L or stimulated Tg ≥ 10 μg/L or TgAb tended to increase and show negative imaging. All imaging results were interpreted by two and more physicians who analyzed the films together. The final treatment response after ^131^I treatment was categorized as follows: the ER group and the NER group (including IDR, SIR, and BIR), and the regression of both groups was analyzed.

### Statistical analyses

SPSS 26.0 software was applied to statistically analyze the data. The measurement data that conformed to normal distribution were expressed as*¯χ* ± s and those that did not conform to normal distribution were expressed as M (*P*_25,_ *P*_75_); the count data were presented as a rate or frequency. Two independent *t*-samples were employed to compare the age gap between the two groups; the *χ^2^* test was adopted to compare the gender, multifocal tumor, bilateral tumor, stage, and lymph node location of the two groups; the Mann–Whitney *U-*test was applied to analyze the differences in the maximum tumor diameter, the number of metastatic lymph nodes, metastatic lymph node size, metastatic lymph node ratio, ps-Tg level, and 24-h ^131^I uptake rate between the two groups. The receiver operating characteristic (ROC) curve was utilized to determine the value of ps-Tg in predicting the achievement of ER after ^131^I treatment. Multifactorial logistic regression was applied to investigate independent factors that affect ER. The results of *BRAF^V600E^* test in different two treatment groups were analyzed by *χ^2^* test. And a statistically significant distinction was deemed to exist at P < 0.05.

## Results

1. General Information Comparison. A total of 175 participants with intermediate-risk PTC were enrolled in this survey, with 123 (70.3%, 123/175) and 52 (29.7%, 52/175) subjects assigned to the ER and NER groups, respectively, which included 17 (9.7%, 17/175) in SIR, 22 (12.6%, 22/175) in IDR, and 13 (7.4%, 13/135) in BIR. Statistically considerable variations in the maximum tumor diameter (*U*=2495.50, *P*=0.021), the number of metastatic lymph nodes (*U*=2313.50, *P*=0.004), the size of metastatic lymph nodes (*U*=2113.50, *P*=0.003), metastatic lymph node ratio (*U* = 2111.50, *P* = 0.004), metastatic lymph node location (*χ^2^* = 9.20, *P* = 0.002) and ps-Tg level (*U*=1011.00, *P<* 0.001) between the two groups. The remaining indexes (i.e., age, gender, tumor multifocality, tumor bilaterality, 24-h ^131^I-uptake rate, and stage) were not statistically significant between the two departments (P > 0.05; **Table 1**).

**Table 1:** Comparison between two divisions of individuals with papillary thyroid cancer in terms of general clinical data.

	Clinical regression		Test value	P
	ER	NER		
Age ( $\bar{\text{x}}$ ± s, years)	41.0 ± 10.4	44.5 ± 12.2	-1.925^a^	0.056
Sex				
Male	39	19	0.385^b^	0.535
Female	84	33		
Maximum tumor diameter	0.8 (0.5, 1.2)	1.1 (0.6, 1.5)	2495.50	0.021
tumor multifocality				
Single spot	84	29	2.506^b^	0.113
multiple spots	39	23		
tumor bilaterality				
Unilateral	90	32	2.342^b^	0.126
Bilateral	33	20		
Stage (AJCC 8^th^) [M(P_25_, P_75_)]				
I	108	42	3.273^b^	0.195
II	15	9		
III	0	1
number of metastatic lymph nodes [M(P_25_, P_75_)]	3 (2, 5)	4.5 (2, 11.8)	2313.50	0.004
size of metastatic lymph nodes [M(P_25_, P_75_)]	0.5 (0.3, 0.6)	0.4 (0.6, 1.2)	2113.50	0.003
ps-Tg (ug/L) [M(P_25_, P_75_)]	1.9 (0.5, 4.5)	13.3 (4.0, 32.4)	1011.00	0.000
Metastatic lymph node ratio (%) [M(P_25_, P_75_)]	50 (30, 70)	70 (50, 90)	2111.50	0.004
Metastatic lymph node location				
central neck	95	29	9.20^b^	0.002
Lateral neck	23	21		
24-h ^131^I uptake rate [M(P_25_, P_75_)]	2.5 (1.3, 4.3)	2.1 (1.0, 3.9)	2891.00	0.316

_a_t value, ^b^χ2 value, the remainder is U value; ER was satisfactory, and non-ER included IDR, BIR, and SIR. Ps-Tg was stimulant thyroglobulin before ^131^I treatment. Staging in conformity with the American Joint Committee on Cancer AJCC 8^th^ edition.

2. Multivariate regression analysis. In the multifactorial logistic regression analysis, clinical regression was considered as the dependent variable, while using ps-Tg, metastatic lymph node size, the number of metastatic lymph nodes, maximum tumor diameter, the location of metastatic lymph nodes, and metastatic lymph node ratio were included in the multi-categorical logistic regression equation, with P < 0.05 in the univariate analysis considered as independent variables. The outcomes revealed that Ps-Tg (OR = 1.209, 95% *CI*: 1.120–1.305, *P<* 0.001) were used as independent factors affecting ER; maximum tumor diameter (OR = 0.927, 95% CI. 0.473–1.817, P = 0.826), the number of metastatic lymph nodes (OR = 1.089, 95% CI: 0.957–1.239, P = 0.198), metastatic lymph node size (OR = 1.527, 95% CI: 0.481–4.852, P = 0.473), the location of metastatic lymph nodes (OR = 0.494, 95% CI: 0.155–1.580, P = 0.235), and metastatic lymph node ratio (OR = 1.374, 95% CI: 0.229–8.255, P = 0.728) could not be used as independent factors affecting ER (**Table 2**).

**Table 2:** Results of multifactorial logistic regression analysis affecting ER in patients with intermediate-risk PTC.

Factors	B	SE	Wald	OR	95%CI	P
ps-Tg	0.190	0.039	23.753	1.209	1.120–1.305	0.000
Metastatic lymph node size	0.424	0.590	0.516	1.527	0.481–4.852	0.473
Number of metastatic lymph nodes	0.085	0.066	1.657	1.089	0.957–1.239	0.198
Maximum tumor diameter	-0.076	0.343	0.049	0.927	0.473–1.817	0.826
Metastatic lymph node location	-0.704	0.593	1.411	0.494	0.155–1.580	0.235
Metastatic lymph node ratio	0.318	0.915	0.121	1.374	0.229–8.255	0.728

**Figure 1:**
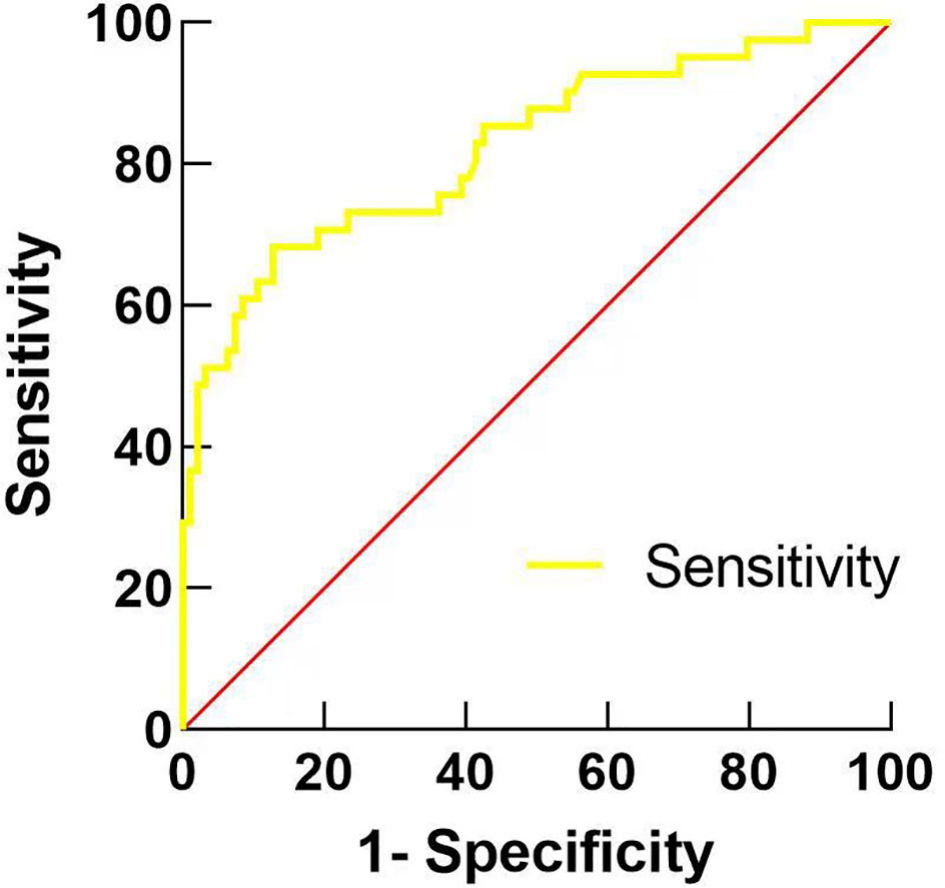
ROC curve of stimulated thyroglobulin (ps-Tg) before the first ^131^I treatment to predict the best treatment response in 175 patients with intermediate-risk PTC.

3. ROC curve analysis. The cut-off value of ps-Tg for predicting ER in intermediate-risk patients was 6.915 ug/L, with the maximum Jorden index of 0.586 and an area under the curve of 0.842 (95% CI: 0.774–0.910), which corresponded to a sensitivity and specificity of 69.2% and 89.4%, respectively (**Figure 1**).

4. Relationship between *BRAF^V600E^* and efficacy. A total of 95 patients were tested for the *BRAF^V600E^* gene in this study, which included 11.6% cases of wild-type *BRAF^V600E^* (11/95) and 88.4% cases of mutant *BRAFV600E* (84/95). The two groups did not differ significantly from each other (*χ^2 =^* 0.931, *P* = 0.335).

## Discussion

In this study, we investigated the clinical regression of intermediate-risk PTC patients after their first ^131^I treatment, with 70.3% of subjects achieving ER based on real-time dynamic assessment, considering the extremely low rate of disease recurrence and the associated mortality in the ER state (1–4%; disease-specific death occurrence in <1% cases) (7). Irrespective of their initial risk classification, PTC patients who experienced clinical remission after the initial treatment showed a decreased probability of long-term relapse (9). Finally, the follow-up was appropriately scaled back in terms of both intensity and frequency, and the treatment target of TSH inhibition was relaxed. However, 29.7% of the patients still did not reach the ER state (namely, the NER state), which necessitates closer monitoring and greater attention.

Tg is a homodimeric macromolecular glycoprotein secreted by the thyroid follicular epithelial cells, which is intimately correlated with postoperative residual thyroid tissue and/or residual tumor tissue along with remission, persistence, recurrence, or distant metastasis of disease after the initial treatment, and can be considered as an essential indicator for monitoring tumor recurrence and metastasis (10). Based on the study conclusion, after undertaking the ^131^I therapy, the NER group’s ps-Tg levels were considerably greater than those of the ER group, which suggests that those with low ps-Tg levels are more likely to reach the ER state. In the present study, the ps-Tg levels were discovered to act as an independent predictive indicator for the outcome of ^131^I treatment, which is consistent with the conclusions of past reports (11–13). Although postoperative serum Tg level can be a useful indicator of the possibility of remission, persistence, or recurrence of disease after the initial therapy, its predictive usefulness is affected by several variables, which include the duration after total thyroidectomy, residual thyroid tissues, and the serum TSH and TgAb levels (14, 15). Moreover, there is no clear optimal ps-Tg cut-off point established yet for the prediction of ^131^I therapeutic outcomes.

Further analysis by ROC curves demonstrates that the ps-Tg cutoff value of 6.915 ug/L had a significant predictive value for the clinical regression of patients. The ps-Tg cut-off values for forecasting the outcome of the first ^131^I treatment for DTC in past trials were 3.8–10 ug/L (1, 11–13, 16, 17). A retrospective analysis revealed that the ps-Tg level <8.55 ug/L before ablation predicted 88% sensitivity and 72% specificity for disease remission following 18–24 months of ^131^I therapy (18). Zheng et al. (19) demonstrated that the ps-Tg level <9.51 ug/L indicated better efficacy of ^131^I treatment in patients with DTC. The present study found that a cut-off value of 6.915 ug/L predicted ER, which is similar to the results of the abovementioned past studies.

When compared with past studies, the following differences were recorded. First, most of the early studies focused on the success of nail removal as the goal of the first ^131^I treatment; however, in this study, clinical regression was set as the goal of treatment. Second, the patients in past research displayed multiple DTC subtypes, whereas, in this study, it was restricted to patients with PTC, which minimized the heterogeneity induced by distinct pathological aspects of the DTC subtypes. Third, the included study population was different from that in the previous study in terms of the difference in the risk stratification; this study focused exclusively on intermediate-risk patients. As a result, the present study findings can facilitate physicians by predicting the clinical regression of intermediate-risk patients after treatment based on the sTg value in advance. Notably, the sTg cut-off value is relevant to the time of Tg measurement. For instance, Heemstra (20) demonstrated that the sensitivity and specificity of the same cut-off value varied at different time points, while the highest diagnostic accuracy of serum ps-Tg level for tumors was during TSH-stimulation, namely 6 months after the initial treatment (21). Furthermore, whether more aggressive treatment such as an appropriate increase in the ^131^I treatment dose can be administered to PTC patients with ps-Tg level >6.915 ug/L warrants further exploration.

In this study, univariate analysis revealed that the maximum tumor diameter, the number of metastatic lymph nodes, metastatic lymph node size, the location of metastatic lymph nodes, metastatic lymph node ratio, and the ps-Tg level were all significantly associated with clinical regression after ^131^I treatment. Past studies have demonstrated that the tumor diameter is closely related to DTC recurrence and metastasis, with a greater tumor diameter indicating a relatively worse prognosis (22). PTC recurrence was predicted by a tumor size of >4 cm (23). It has also been established by several studies that the greater the number of lymph node metastases, the worse the clinical outcome (24, 25), and the local recurrence rate of DTC patients gradually increases with an increase in the number and diameter of lymph node metastases (26, 27). In support, several researchers have reported that ^131^I has a significant efficacy when the maximum diameter of the lymph node is <1 cm (28). Furthermore, the lymph node ratio has been proposed as a valuable prognostic indicator for PTC (28). In their large sample study, Amit et al. (29) demonstrated that the metastatic lymph node ratio can be used as a predictor of 10-year overall and disease-specific survival. According to the results of De Meer et al. (1), individuals with metastatic lymph nodes in the lateral cervical region experienced a much higher rate of recurrence than those with metastases in the central region. It has also been reported that the residual thyroid tissues before ^131^I treatment and the ^131^I uptake rate of residual thyroid tissues affect the efficacy after ^131^I treatment and that there exists a close relationship between these factors. Generally, the fewer residual thyroid tissues after surgery, the lower the ^131^I uptake rate of residual thyroid tissue. Presently, the residual thyroid tissue can be determined by neck color ultrasound and thyroid radionulid imaging, although it is difficult to accurately calculate its actual mass. Therefore, the residual thyroid tissue before ^131^I treatment was not included in the present analysis, although the ^131^I uptake rate, which is closely related to the residual thyroid tissues, was assessed. No statistically significant difference was recorded between the groups in terms of age, gender, tumor multilaterality, tumor bilaterality, 24-h ^131^I uptake rate, or the tumor stage.

The most frequent genetic mutation in PTC is that of *BRAF^V600E^*, which results in aberrant cell growth and carcinogenesis *via* the MAPK/ERK pathway (30). The *BRAF^V600E^* mutant has been associated with increased pathological invasiveness, recurrence, as well as mortality (31, 32) from primary tumors in a series of research. Past reports have linked the *BRAF^V600E^* mutation to aggressive behavior and a poor prognosis in PTC (33). Furthermore, it has been reported that *BRAF^V600E^* acts as a reliable predictor of recurrence risk in PTC patients although there are conflicting viewpoints reported in the sections of literary works. Some researchers have discovered that the *BRAF^V600E^* mutation is not related to more widespread or aggressive clinical and pathological characteristics (such as extrathyroidal invasion, lymph node metastasis and so on) and is, therefore, not predictive of recurrence or survival in PTC (34–36). In fact, *BRAF^V600E^* was insufficient in affecting the clinical outcome in the present study. Thus, in terms of the prognostic value, *BRAF^V600E^* mutation in PTC remains elusive, although its utility may vary with the study population (36).

Multifactorial analysis revealed that ps-Tg level can act as an independent factor influencing the outcome of ^131^I treatment. In this study, the results of maximum tumor diameter and the quantity, size, location, and ratio of metastatic lymph nodes, as well as the ps-Tg level were all determined to be significant factors as per the results of univariate analysis, but not as per those of multivariate analyses. This finding indicates that these factors alone did not affect the outcome of ^131^I treatment. Therefore, we speculated that the combination of these factors, and not individually, may have influenced the outcome of ^131^I treatment.

This study has some limitations. As this was a retrospective cohort study, selection bias could not be ruled out. Second, the number of study subjects enrolled was relatively small due to incomplete case data and lost visits. Third, owing to the short follow-up period of this study, a longer monitoring period is warranted, considering the inert nature of PTC.

## Conclusion

In summary, patients with intermediate-risk PTC and smaller tumors, a lower metastatic lymph node ratio, more centrally located metastatic lymph nodes, fewer metastatic lymph nodes, smaller metastatic lymph node diameters, and ps-Tg levels <6.915 ug/L were more likely to achieve ER after the initial treatment. Based on these influencing factors, there seems to be a certain predictive value for clinical regression after ^131^I treatment in intermediate-risk PTC patients, and the frequency and intensity of monitoring get reduced during the follow-up to timely adjust the treatment regimen, reduce the economic burden on the patients, and provide an auxiliary basis for further individualized and precise treatment.

## Data availability statement

The original contributions presented in the study are included in the article/**Supplementary Material**. Further inquiries can be directed to the corresponding author.

## Author contributions

YL, MR, and GY edited the manuscript. CZ collected samples. JH and DF analyzed data. YX created the figures. GY designed and coordinated the work. All authors contributed to the article and approved the submitted version.

## Conflict of interest

The authors declare that the research was conducted in the absence of any commercial or financial relationships that could be construed as a potential conflict of interest.

## Publisher’s note

All claims expressed in this article are solely those of the authors and do not necessarily represent those of their affiliated organizations, or those of the publisher, the editors and the reviewers. Any product that may be evaluated in this article, or claim that may be made by its manufacturer, is not guaranteed or endorsed by the publisher.

## Supplementary material

The Supplementary Material for this article can be foundonline at: https://www.frontiersin.org/articles/10.3389/fendo.2022.1015798/full#supplementary-material
